# Evaluating group dynamics through peer assessment during a global student collaboration of interprofessional healthcare education: A longitudinal study across 33 universities

**DOI:** 10.1002/ase.70026

**Published:** 2025-03-27

**Authors:** Mandeep Gill Sagoo, Pak Yin Lam, Tanvi Sharma, Arisma Arora, Maheen Siddiqui, Adedeji M. Adeniyi, Cecilia Brassett, Geoffroy Noel, Richard Wingate, Sean McWatt, Dana Stearns, Pilar Garcia Souto, Anette Wu

**Affiliations:** ^1^ Faculty of Life Sciences and Medicine King's College London London UK; ^2^ College of Dental Medicine Columbia University New York New York USA; ^3^ Vagelos College of Physicians and Surgeons Columbia University New York New York USA; ^4^ Department of Medicine University of Cambridge Cambridge UK; ^5^ Department of Surgery University of California San Diego La Jolla California USA; ^6^ Schulich School of Medicine and Dentistry Western University London Ontario Canada; ^7^ Department of Emergency Medicine Massachusetts General Hospital Boston Massachusetts USA; ^8^ Department of Medical Physics and Biomedical Engineering University College London London UK

**Keywords:** collaboration, global healthcare, group work, interprofessional

## Abstract

With the advent of healthcare globalization, interprofessional collaboration has become increasingly important on an international scale. This longitudinal study evaluated group dynamics in the *International Collaboration and Exchange Program*, a global online program of students across 33 universities from diverse healthcare backgrounds, including medicine, dentistry, pharmacy, and biomedical science. In groups of 4 to 6, participants engaged in regular discussions and projects relating to anatomy education and global health. Peer assessment was used to determine (1) whether a relationship existed between group cohesiveness and disparities in individual contribution levels and (2) whether group cohesiveness and individual contribution levels changed over time across varying group sizes. Two student cohorts were studied using the Individual Peer Assessment of Contribution methodology. Peer assessment surveys were distributed at two time points for the first (2021–2022) and second (2022–2023) cohorts, respectively, yielding 423 responses from 126 groups. Collaboration quality and effectiveness were evaluated through numerical ratings and qualitative feedback. Peer assessment is a viable tool for evaluating the dynamics of group interactions in virtual collaboration on a global scale. A reduction in group cohesiveness was associated with greater imbalances in individual contribution levels (*r* = −0.71, *p* < 0.001). Furthermore, larger groups (*n* = 6 students) demonstrated improved cohesiveness and equality in individual contribution levels over time compared to smaller groups (*n* = 4 students). This study on international healthcare student collaboration provides insights into sociocultural and educational factors impacting virtual group interactions and offers strategies for enhancing interprofessional collaborative practices in global health education.

## INTRODUCTION

As healthcare globalization increases in prevalence, the importance of interprofessional collaboration and education is rising concomitantly on an international scale. However, opportunities for interprofessional education (IPE) among healthcare students remain limited globally, and the study of teamwork and group interactions between healthcare students across geographical and sociocultural borders remains largely unexplored. This study evaluates the group dynamics of virtual student collaboration by implementing peer assessment in an international cohort of healthcare students. The study aims to contribute useful information on the nature of group interactions on a global scale and lay the groundwork for developing IPE teaching strategies and frameworks to address the challenges posed by multicultural and multidisciplinary collaboration in global healthcare teams.

### Interprofessional collaboration and education

Interprofessional collaboration occurs when two or more professions work together to solve complex problems in the pursuit of common goals, with shared responsibility and mutual authority.^1^ With the advent of healthcare globalization, the need for interprofessional collaboration extends to the international arena. Advances in telemedicine, global health challenges (e.g., the spread of infectious diseases, pandemics, and chronic illnesses), and rapid market expansion of healthcare providers across borders collectively fuel the need for interdisciplinary collaboration in healthcare provision across countries.^2^, ^3^, ^4^ Additionally, as patient populations become more culturally diverse, promoting cultural awareness among healthcare professionals becomes increasingly important.^5^, ^6^ International interprofessional collaboration is essential to facilitating cultural awareness and enhancing communication with diverse patient populations.

Successful interprofessional collaboration begins with interprofessional education (IPE).^4^, ^7^ However, in spite of growing opportunities for IPE among existing healthcare professionals, its integration into educational curricula for healthcare students remains limited globally. This could be due to a lack of support from universities, logistical challenges, and/or inadequate staff development.^8^, ^9^, ^10^, ^11^ IPE in tertiary education is crucial to enhancing students' understanding and appreciation of various healthcare disciplines, building mutual respect, facilitating communication and team‐building skills, and raising cultural awareness and competence early in their training.^12^ In anatomy education, international IPE further provides a unique platform for students to share how anatomy is taught in different countries, including their experiences in donor dissections, ethical and cultural perspectives on body donations, donor management, and reflections on the topic of death.^13^ Such collaborations also encourage sharing diverse teaching methods and formats and provide valuable insights into how anatomy curricula can be improved globally.

### The international collaboration and exchange program

Group work is an integral component of IPE. Bringing students together to work in groups facilitates close collaboration and communication among future healthcare professionals.^14^, ^15^ This can be done through structured group activities such as group discussions, case studies, platform debates, and projects that require students to apply their collective knowledge and skills to solve complex healthcare challenges.

In view of the need to promote IPE through group work internationally, the *International Collaboration and Exchange Program* (ICEP) was established in 2014, as a large‐scale online networking program bringing together medical and health professions students from across the globe to promote networking, the sharing of ideas, and engagement in internationally collaborative research and education.^16^ Originally initiated by Columbia University from within its anatomy course, ICEP now partners with 33 leading medical universities across Europe, America, Asia, and Australia, connecting over 400 students in medicine, dentistry, pharmacy, and the biomedical sciences. Through weekly online group discussions, students share their learning experiences in anatomy education, including dissections, prosections, medical imaging, and healthcare systems in their respective countries, and discuss ethical issues in global health.

This longitudinal study investigates the group dynamics of student collaboration within global healthcare student cohorts in ICEP, offering healthcare professionals and educators a deeper understanding of the cultural nuances, values, and communication styles that influence interprofessional interactions in IPE on a global scale. International collaboration in IPE presents unique challenges, such as sociocultural diversity, language barriers, and disparities in educational backgrounds. Analyzing how group dynamics change over time results in a deeper understanding of how these factors affect group interactions, peer contributions, and collective decision‐making, informing future teaching strategies for IPE that promote cultural awareness, experiential learning, and leadership development.

### Evaluating group dynamics through peer assessment

Group dynamics can be broadly measured in terms of two aspects: group cohesiveness and individual contribution levels. Group cohesiveness is defined as “the resultant forces acting on members to stay in a group”,^17^ referring to the unity and sense of belonging between group members. Individual contribution reflects how evenly the workload is shared. Significant disparities in contribution, such as “*free riders*” contributing less, can undermine trust and cohesion within the group, causing resentment and frustration.^18^, ^19^, ^20^

One approach to evaluate group dynamics in IPE is through *peer assessment*, where students assess the performance of fellow group members via peer feedback.^21^, ^22^ This may take the form of numerical ratings (e.g., on a Likert scale) or written comments. Positive feedback indicates high group cohesiveness, with a strong degree of mutual trust, respect, and support. Conversely, large discrepancies in feedback within a group indicate potential imbalances in contributions between group members.

This study used peer assessment to evaluate the group dynamics of international healthcare student cohorts in ICEP. This study aimed to (1) assess whether an association exists between group cohesiveness and disparities in individual contribution levels, (2) determine how group cohesiveness and individual contribution levels change over time and whether group sizes influence these changes, and (3) explore the qualities that constitute a productive and sustainable group dynamic through qualitative analysis of peer comments from participating students.

## METHODS

Ethics approval was obtained through the Research Ethics Management Application System (REMAS) for King's College London (approval number MRA‐21/22‐26348), and through the Institutional Review Board (IRB) of Columbia University (approval number AAAO3715).

### Group work in the international collaboration and exchange program (ICEP)

ICEP is a global year‐long program that runs annually from October to May, with the aim of fostering international leadership, collaborative problem‐solving skills, and cultural competence among healthcare students from an early stage of their training. The program brings together students from 15+ countries (Figure S1) from diverse educational backgrounds in medicine, dentistry, pharmacy, and biomedical science.

During the program, participants are randomly divided into groups of four, five, or six students. The random allocation ensures similar geographical and educational diversity among students in each group. Throughout the year, students organize weekly group meetings online to discuss topics related to anatomy education, global healthcare, or medical ethics and law. Past discussion topics have included historical and cultural perspectives on body donation and donor dissections, incorporating digital technologies and virtual reality in anatomy education, and differences in public healthcare systems between countries. Weekly small‐group discussions are supplemented by monthly online sessions on Zoom™ (Zoom Video Communications, Inc.) involving the entire student cohort, consisting of large‐group platform debates and seminars with guest speakers from around the world. At the end of the program, each group is tasked with creating a video presentation or submitting a poster or literature review essay on a topic relating to anatomy education, medical ethics, or global healthcare issues. A group leader is elected by members of each group at the start of the program; however, there is no specific framework for assigning definitive roles to other members. Students are free to decide how to distribute the workload and responsibilities in group projects and discussions throughout the program.

### Data collection from student surveys

This study evaluated two student cohorts in ICEP: 2021–2022 (21–22) and 2022–2023 (22–23). Students were invited to complete a peer assessment survey on two occasions throughout the program: the first round (Round 1) in December 2021 and November 2022 for the 21–22 and 22–23 cohorts, respectively, and the second round (Round 2) in March 2022 and February 2023. In the survey, students were asked to give a rating to every group member based on their performance and professional behavior in group work, in accordance with each of the following assessment attributes: (1) overall rating, (2) quality of work and insightful ideas, (3) punctuality at group meetings, (4) respect shown to team members, and (5) leadership skills. Each rating is given as a number between 1 (lowest) and 5 (highest). The full survey and rating criteria are presented in Table S1.

Surveys were collected and evaluated using the Individual Peer Assessment of Contribution (IPAC) methodology.^23^ This methodology was initially developed as an alternative assessment strategy that incorporates peer assessment into the marking/grading of group work. Peer ratings were used to calculate a score that was used as an adjunct to group assessments to determine each student's final score.^23^ Variations of this methodology have been proposed and implemented in local student cohorts for small‐group projects, including by Conway et al. in an optometry course at Hong Kong Polytechnic University,^24^ and by Northrup et al. in an undergraduate engineering course at Western New England College.^25^ In 2015, the *IPAC System*, a software developed by University College London, was the first to standardize, streamline, and automate this grading process.^26^ The software allows fully automated processing of peer feedback and calculation of student scores, enabling a sustainable and time‐efficient implementation of the IPAC methodology on a larger scale.^27^, ^28^

Both the peer assessment survey and the IPAC software have been trialed in several published studies by University College London.^23^, ^27^, ^29^ It has since been used by educators at University College London to assess group projects across various class sizes, and it has been well received by both staff and students. More recently, it was adapted by the Synthetic Anatomy module at King's College London.^30^ This study implemented the peer assessment survey and IPAC software in an international student cohort. Evaluating the distribution of peer assessment scores is intended to shed light on the dynamics of group work within each student group on a global scale.

Additionally, students were given the opportunity to provide written feedback on the performance of each group member. To preserve anonymity and avoid potential bias, students could not see what others had written; this ensured that they could provide honest evaluations without concern for how others may perceive their feedback. Participation in this study and submission of peer assessment surveys was entirely voluntary. The survey was designed using Qualtrics, and responses were collated using Microsoft Excel.

### Quantitative evaluation of group dynamics using the individual peer assessment of contribution methodology

Group cohesiveness and individual contribution levels were evaluated using data obtained from the peer assessment survey. In accordance with the IPAC methodology, deidentified ratings received by each student were used to calculate an IPAC score in the form of (1) a percentage or (2) a normalized factor. The percentage (%) IPAC score was calculated by taking the mean rating received as a percentage of the maximum possible rating that could be received (5 in this case), and ranges between 0% and 100% (Equation 1). A high mean percentage IPAC score indicates high group cohesiveness. The normalized IPAC score was calculated by taking the mean rating received by the student and dividing it by the overall average rating received by group members (Equation 2). A normalized score >1 indicates that the student has received a higher rating than the rest of the group, suggesting a greater contribution to group work. In contrast, a normalized score of <1 indicates that the student has received a lower rating, suggesting that the student has contributed less. Therefore, if normalized scores are widespread within a group (i.e., with a large standard deviation and variance), this indicates a disparity in individual contribution levels among its members.

(1) $$\%\text{IPAC score}:P=\frac{\text{Mean rating received}}{5}\times 100\%,0\leq P\leq 100$$

(2) $$\begin{array}{c} \text{Normalized IPAC score}:N=\frac{\text{Mean rating received}}{\text{Mean rating received}\;\mathrm{by}\;\mathrm{all}\;\text{members}} \end{array}$$

Distributions of percentage and normalized IPAC scores were analyzed using Microsoft Excel, IBM SPSS Statistics (version v29), and R (version 4.4.1). The internal consistency of the peer assessment survey's Likert‐scale section was evaluated using Cronbach's alpha to ensure reasonable confidence in interpreting the survey's results and the downstream outcomes reliant on the survey's data. Cronbach's alpha was interpreted according to DeVellis' parameters: <0.600 unacceptable; 0.600–0.649 undesirable; 0.650–0.699 minimally acceptable; 0.700–0.799 respectable; 0.800–0.899 very good; ≥0.900 excellent.^31^

To investigate the association between group cohesiveness and individual contribution levels, the standard deviation of normalized IPAC scores was plotted against the average percentage IPAC score for each group, and a Pearson's correlation coefficient (*r*) was calculated to assess the linearity of their relationship. Statistical significance was determined by obtaining its corresponding *T*‐statistic. Furthermore, to investigate changes in group cohesiveness and levels of individual contribution over time, average percentage IPAC scores and normalized IPAC score distributions were compared between Rounds 1 and 2 of the programs for groups of four, five, and six students, respectively. Statistically significant differences in average percentage IPAC scores were determined using a paired *t*‐test. The effect size was measured using Cohen's *d*, which was interpreted as small (*d* < 0.2), medium (*d* = 0.2–0.5), or large (*d* > 0.5).^32^ Significant differences in correlated variance of normalized IPAC scores were determined using the Pitman‐Morgan test.^33^, ^34^ The significance level, alpha, was set at 0.05.

### Qualitative evaluation of group dynamics through written feedback

Finally, qualitative content analysis was conducted on free‐text peer feedback from students. Two independent authors performed this analysis using manual thematic coding.^35^ Discrepancies were resolved by a third independent author. The codes extracted from student comments included informative contributions, active leadership, inclusivity and active listening, commitment and resilience, lack of participation, coordination among group members, and consideration for others. These codes were arbitrarily assigned numbers 1–6. Emergent themes were identified using an inductive approach. Cohen's Kappa showed “almost perfect agreement” between raters (*κ* = 0.84 ± 0.081).^36^

## RESULTS

A total of 423 peer assessment survey responses from 126 groups were received in this study. In the 21–22 cohort, 244 responses were received in Round 1 (December 2021) and 154 in Round 2 (March 2022). In the 22–23 cohort, 179 responses were received in Round 1 (November 2022) and 55 in Round 2 (February 2023). Overall distributions of percentage and normalized IPAC scores from Rounds 1 and 2 across both cohorts are presented in Figure S2. Comparisons of IPAC score distributions with previous studies are presented in Tables S2 and S3.^24^, ^25^, ^27^, ^37^, ^38^, ^39^ Cronbach's alpha values showed an internal consistency of “excellent” for the survey across all cohort responses (Table S4).

### Increased group cohesiveness is associated with reduced disparities in individual contribution levels

**FIGURE 1:**
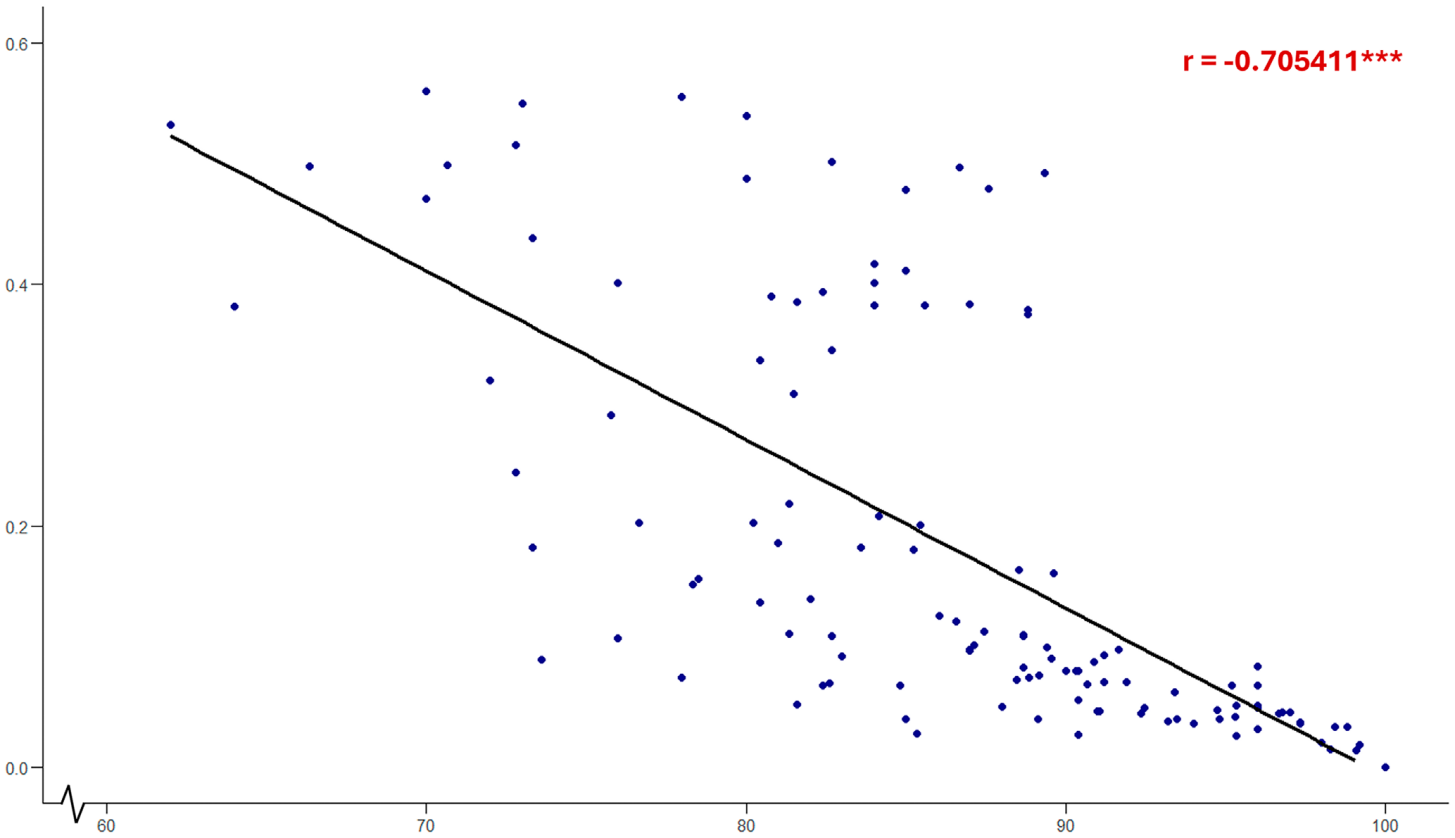
Scatter plot of standard deviations of normalized IPAC scores against average percentage IPAC scores of each group (*n* = 126). ***p < 0.001.

Standard deviations of normalized IPAC scores and average percentage IPAC scores were plotted on a scatter graph, as shown in Figure 1. A large negative correlation was observed (*r* = −0.71, *p* < 0.001). This indicates that differences in individual contribution levels decrease between group members as group cohesiveness increases.

### Larger groups collaborate more effectively over time compared to smaller groups

Table 1 shows the changes in average percentage IPAC scores for groups of four to six students between Rounds 1 and 2, covering a span of 4 months. A statistically significant decrease was observed in four‐student groups, with a medium effect size (*p* < 0.05, *d* = −0.522). No significant change was observed in five‐student groups, and a significant increase was observed in six‐student groups, with a small effect size (*p* < 0.05, *d* = 0.290).

**TABLE 1:** Average percentage IPAC scores between Rounds 1 and 2 for four to six‐student groups.

	Round 1	Round 2	Change
4‐student groups	87.40	79.46	−7.94*
5‐student groups	87.97	88.79	+0.82
6‐student groups	84.49	88.58	+4.09*

* *p* < 0.05.

**FIGURE 2:**
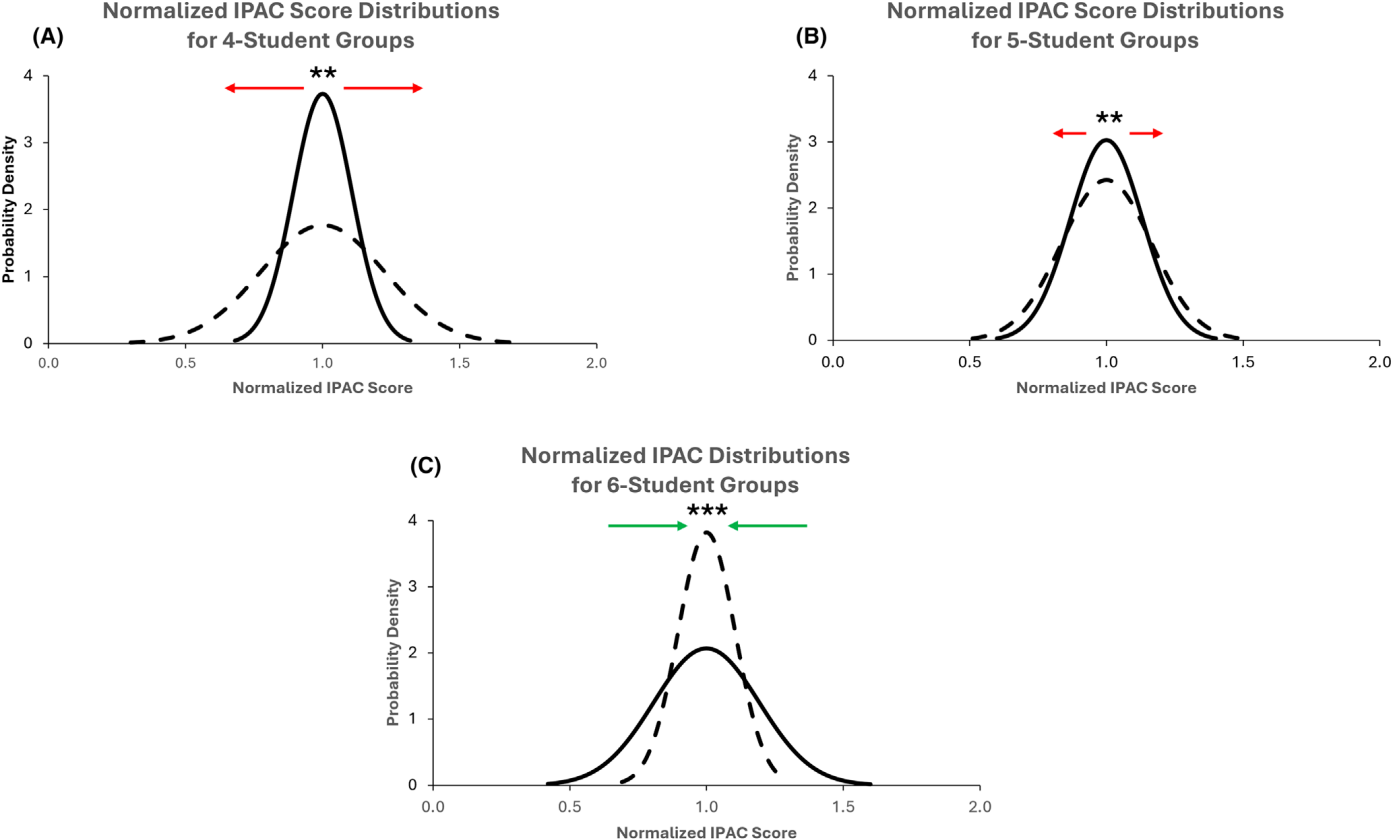
Normalized IPAC score distributions between Round 1 (solid line) and Round 2 (dashed line) for four‐student groups (A), five‐student groups (B), and six‐student groups (C). **p < 0.01, ***p < 0.001.

Changes in normalized IPAC score distributions for groups of four to six students between Rounds 1 and 2 are shown in Figure 2. Score distributions of groups with four students became significantly more widespread over time (*p* < 0.01), with an associated significant increase in standard deviation and variance. Score distributions of groups with five students also increased over time but to a lesser extent (*p* < 0.01). However, the opposite was observed for groups with six students, whereby the distribution became significantly narrower in Round 2 (*p* < 0.001). Collectively, these results demonstrate a trend in which larger (six‐student) groups show stronger group cohesiveness and equality in individual contribution levels over time, while smaller (four‐student) groups exhibit a gradual deterioration in cohesiveness and greater imbalance in individual contribution levels over time.

### Thematic analysis of student comments

Qualitative analysis of student comments highlighted two overarching themes in sustainable teamwork: individual engagement and leadership skills (Table 2). Students who attained higher ratings from their peers tended to receive comments relating to enthusiasm and participation in group activities and actively contributed ideas during discussions. Some students received further praise for attending group meetings in spite of time zone differences. In contrast, students who were less engaged in group discussions received lower peer ratings. Leadership and organizational skills were also a prevalent theme. Comments from groups with high cohesiveness highlighted the importance of the group leader's role in facilitating group discussions, time management, and critical decision‐making.

**TABLE 2:** Sample feedback from students under each theme.

Theme	Code	Sample comments
Engagement in group work	Informative contributions	“Contributes well thought‐out and informative points about their own experiences with the healthcare system in their country”
	Commitment and resilience	“[Student] was very committed, attending group meetings even when [student] had to wake up at 5 am due to time zone differences” “Makes an effort to attend the majority of group discussions in spite of technical difficulties, which shows resilience and dedication to the project” “Tries their best to be available for meetings even with very busy schedules and time zone difficulty”
	Lack of participation	“[Student] was really nice and contributed interesting ideas at the start, but essentially ghosted us in February. We have not heard from [student] since, and our messages have been left on read. This was honestly quite unpleasant” “[Student] is no longer considered part of our group. [Student] did not respond to any messages, did not participate in the last group meeting and did not contribute to our final project presentation”
Leadership and organizational skills	Active leadership	“Takes charge in the direction and clarity of discussions, demonstrating great analytical and problem‐solving skills” “Helped organise most of our group activities and kept our team heading toward the right direction”
	Inclusivity and active listening	“Always listens to everyone's ideas before giving their own input, offering solutions to any points of discussion that we were unsure about” “Very conscious about others' thoughts and feelings. Makes sure to stay respectful to others and invite them to contribute” “Facilitates group discussions and encourage others to speak when they haven't spoken much”
	Coordination and consideration for others	“Really great at coordinating the group… one of the members had been skipping meetings, and he was the one to call them out” “A great team player and has been a key person in shaping a positive and animated team atmosphere” “Actively takes charge but also creates space for every one of our group members to freely share their thoughts without judgement” “Organizes meetings well and is considerate of everyone's schedules. Makes an effort to engage everyone in meetings even though English was not their first language”

## DISCUSSION

This study investigated the group dynamics of global interprofessional education among university students from various healthcare disciplines in terms of group cohesiveness and levels of individual contribution via the implementation of the IPAC methodology. In general, percentage IPAC scores followed a one‐tailed distribution, with over half of students receiving a score of over 80%. Normalized scores demonstrated a normal Gaussian distribution centered around 1, in line with previous studies.^24^, ^28^, ^37^ The absence of extreme values in comparison to the literature^24^, ^25^, ^28^, ^37^, ^38^, ^39^ supports the potential feasibility of the IPAC methodology for peer assessment on a global scale.

### Group cohesiveness and individual contribution in group work

This study found that greater imbalances in individual contribution levels are associated with lower group cohesiveness (Figure 1). This imbalance is likely influenced by a multitude of factors. Students originating from diverse disciplinary backgrounds may possess varying levels of familiarity and experience with interprofessional collaboration, resulting in diverging expectations regarding their roles within the group. This may have led to gaps in communication and synergy, particularly if students felt more aligned with peers from similar healthcare disciplines than others. Furthermore, sociocultural differences in communication styles and approaches to teamwork may have propagated misunderstandings and perceived inequalities in individual contribution levels among team members.^40^ For example, American and European students may be more assertive and outspoken in group meetings. In contrast, students from Southeast Asian backgrounds may have been more reserved, leading to a false impression of reduced engagement in group work. Group dynamics can be further affected by language barriers, particularly among students where English is not their native language or primary medium of instruction at their universities.^41^ Indeed, peer engagement in group meetings was a dominant theme in the written feedback, with some students citing being “shy” and “quiet” as reasons for low peer ratings. Additionally, time zone differences resulted in some students waking up very early or staying up late to attend group meetings with peers from other countries. Time differences, or *temporal distances*, have been argued to be more influential than geographic distances due to consequent changes in work patterns and coordination.^42^

In turn, disparities in individual contribution levels may culminate in a deterioration in group cohesiveness. Students who perceive themselves as carrying a disproportionate share of the workload may develop feelings of resentment and frustration toward their peers. This was evident in the written feedback, where some students complained of having to do extra work as a result of their peers “not responding to messages,” “not submitting their work on time,” or “lack of engagement in group meetings.” Conversely, others who feel marginalized or excluded from group discussions may eventually withdraw from active participation, becoming less motivated to engage in group work.^43^ This generates tension within the group, eroding the mutual trust and collective morale that underpins sustainable cohesion and collaboration. Over time, large disparities in contribution levels can further lead to power struggles and social loafing.^18^, ^43^, ^44^ Group members become less inclined to share their ideas, collaborate on projects, and support each other's efforts as they perceive that their contributions are not recognized equally.

These findings illuminate the unique challenges faced during virtual international collaboration and closely mirror the dynamics of real‐life global healthcare collaboration in many ways. In a multidisciplinary team of diverse educational backgrounds, disparities in individual contribution levels may arise from differences in training and clinical experience, which may be further exacerbated when roles and responsibilities are not clearly defined.^45^ Moreover, sociocultural diversity and language barriers lead to conflicts of ideas, misunderstandings, microaggressions, and stereotyping.^46^ These issues, if left unresolved, may eventually culminate in breakdowns in communication and collaboration, ultimately jeopardizing patient care.

In order to mitigate these challenges to group cohesiveness and individual contribution in interprofessional collaboration, promoting equality and inclusion is paramount in global IPE programs. Small‐group work may be supplemented with cultural competence training through large‐group seminars and discussions on sociocultural values and norms in different countries.^47^, ^48^, ^49^ Workshops on communication techniques and team‐building skills may help imbue students with confidence in open communication, understanding, self‐reflection, and providing constructive feedback to peers.^50^

### Group cohesiveness and individual contribution over time are associated with group size

Our study further found that changes in group cohesiveness and individual contribution over time are influenced by group size. Groups with four students showed a gradual reduction in group cohesiveness and greater disparities in individual contribution levels, while groups with six students showed improved cohesiveness and equality in individual contribution levels over time (Table 1 and Figure 2). These findings contrast previous studies on local student cohorts, which posited that as group size increases, interactions between group members become reduced,^51^, ^52^ resulting in a lack of opportunities to know one another at a personal and social level.^18^ In a study involving undergraduate students, Gentry et al. reported that smaller groups of two to three members worked better than four‐member groups in terms of minimizing group dissension.^51^ In another study involving groups of three to ten students, Bales et al. reported that increased group size is associated with greater discrepancies in participation.^53^ This is complicated by communication challenges such as difficulty arranging in‐person group meetings compared to smaller groups.^18^ In the long term, this leads to deindividualization and feelings of anonymity,^18^, ^20^, ^54^, ^55^ resulting in a higher incidence of social loafing and “free riders”. Nevertheless, research on the relationship between group size, performance, and the nature of group interactions has remained sparse, and several studies involving wider group size ranges have produced inconclusive results.^56^, ^57^, ^58^

A possible explanation for the findings in our study is that four‐student groups simply had fewer students to share the workload compared to six‐student groups. This may precipitate stronger feelings of resentment toward peers who have contributed less, leading to greater perceived disparities in individual contribution levels as they become harsher and stricter in their ratings. On the other hand, groups with more team members tend to distribute the workload and may have experienced less pressure and subsequently enjoyed a better working atmosphere. In addition, the availability of online meeting platforms and social media, which has expanded rapidly during the COVID‐19 pandemic, may have eased communication difficulties originally characteristic of larger groups by alleviating geographical constraints.^59^ The broader distribution of workload is further supplemented by an increased diversity of ideas and perspectives as a result of having more students from various educational and sociocultural backgrounds within the same group.^60^, ^61^ This is evidenced in the written comments (Table 2), where many students from six‐person groups voiced their appreciation for the “invaluable insights” shared by their peers during group discussions on healthcare issues in different countries. In light of the growing interest in collaborative international learning for anatomy and healthcare education,^12^, ^62^, ^63^ findings from this study suggest that virtual collaboration with online platforms provides a unique advantage that enables larger student groups to work together by broadly sharing responsibilities to improve longer‐term sustainability in teamwork.

### Qualities of effective group work in virtual international collaboration

Written comments from students (Table 2) highlighted several key qualities of effective group work in virtual international collaboration. Positive contributions, especially those sharing well‐informed personal experiences, enriched the group's understanding and offered diverse perspectives. Commitment and resilience were also emphasized, with dedicated members overcoming technical challenges and time zone differences. In contrast, disengagement, such as “ghosting,” negatively impacted group morale and performance.

Finally, active leadership and organizational skills were prevalent themes in student comments. This was particularly the case in groups with high cohesiveness, where students praised their group leader's efforts in “scheduling group meetings,” steering the “direction of group discussions,” and “encouraging others to speak when they haven't spoken much.” Leaders who efficiently coordinate meetings ensure high participation and respect from team members and enable each member to serve as a significant contributor to maximize productivity and synergy.^64^, ^65^, ^66^, ^67^ However, *virtual leadership* (i.e., leadership in virtual or digital environments) poses unique challenges in relationship‐building and coordination,^68^, ^69^ and studies on optimal leadership styles for virtual teamwork remain limited and inconclusive.^69^ The impact of various leadership approaches on long‐term group cohesiveness and individual contribution levels in virtual collaboration warrants further investigation.

### Limitations and future directions

This study has several limitations. Firstly, other factors, such as the duration of group work and previous student experiences with group activities, may have had an impact on the dynamics of group interactions that were not explored in this study. To ensure the internal validity of our study groups, a robust selection process was implemented, whereby participants were included based on their voluntary participation and the submission of a proposal. This dual criterion ensured that all selected participants were both willing and adequately prepared to contribute meaningfully to the study, thereby enhancing the overall validity of our findings.^70^ In future studies, different student groups could be assigned projects of varying lengths to investigate the relationship between the duration of group work and group dynamics. A pre‐program questionnaire could also be given to students before starting their group work to evaluate their past experiences with collaborative work.

Language barriers may also influence the perception of engagement, as previously discussed. The impact of language barriers on academic performance, teacher evaluations, and communication between culturally mixed student groups and multinational teams is well documented in the literature.^71^, ^72^, ^73^ The role of students' native language on the perception of individual engagement from peers would be of interest for further investigation in global student cohorts.

Furthermore, the effect of virtual collaboration on the quality of peer‐to‐peer interactions remains poorly understood. There has been concern that online meetings may lead to reduced engagement and concentration, as it is common for members to mute themselves or turn off their cameras when not directly participating in a discussion.^59^ Eye contact and non‐verbal cues that invite contributions, signal agreement, or add emphasis and reinforcement are also difficult to convey in virtual settings, particularly in medium to large groups.^74^ The effects of these contributing factors on long‐term group cohesiveness and individual contribution levels between different group sizes would be of interest for further study in a global context.

Another limitation is that the number of responses received was visibly reduced in Round 2 across both the 21–22 and 22–23 student cohorts, which may have limited the significance and effect size of results. This could be due to a number of students dropping out of the program toward the end due to coursework, examinations, and other personal commitments. Although this cannot be completely mitigated given the voluntary nature of the program, additional support could be provided to participating students who are struggling to maintain both group work in the program and their academic studies, and online reminders could be sent more frequently to encourage as many participants as possible to engage in group discussions, projects, and peer assessments.

Finally, the scope of group sizes examined in this study was limited. There is no universally accepted definition for what constitutes small, medium, and large groups in the context of interprofessional collaboration. The term “group size” itself remains vaguely defined across various studies: some consider groups of four or more members to be small, while others define small groups as having only two or three members.^75^, ^76^ In healthcare, team sizes can vary significantly, ranging from dyadic pairs to multiteam systems.^77^ It would be of interest to further investigate whether the trends observed in our study extend to larger group sizes and other contexts, such as game‐based learning, which is gaining traction for cultivating teamwork and communication skills in health sciences education.^78^

## CONCLUSION

The globalization of healthcare underscores the need to integrate IPE and cultural competence training into healthcare curricula. Promoting IPE at a global level in anatomy education offers students and faculty unique insights into anatomical teaching methods and their cultural perspectives across diverse geographical and social backgrounds. This study investigated group dynamics in international virtual collaboration among a global cohort of healthcare students with diverse geographical and educational backgrounds. The study found that high group cohesiveness is associated with reduced disparities in individual contribution levels over time. Although further studies are needed to establish optimal group sizes for global student collaborations, findings from this study support the unique benefits of online collaboration for effective and sustainable teamwork within larger student groups by overcoming time and geographical barriers, whilst facilitating broader workload distributions and diverse perspectives. Future research should aim to investigate trends in virtual collaborations across a greater range of group sizes. The literature would also benefit from developing and testing tools for measuring students' virtual interactions and learning across diverse settings.

## AUTHOR CONTRIBUTIONS

**Mandeep Gill Sagoo:** Conceptualization; investigation; methodology; validation; writing – review and editing; supervision; resources; project administration. **Pak Yin Lam:** Conceptualization; investigation; writing – original draft; methodology; validation; visualization; software; formal analysis; data curation. **Tanvi Sharma:** Data curation; writing – original draft; validation; formal analysis; writing – review and editing. **Arisma Arora:** Investigation; formal analysis; writing – review and editing. **Maheen Siddiqui:** Writing – original draft; formal analysis. **Adedeji M. Adeniyi:** Writing – review and editing; data curation. **Cecilia Brassett:** Writing – review and editing; supervision. **Geoffroy Noel:** Supervision; writing – review and editing. **Richard Wingate:** Writing – review and editing; supervision. **Sean McWatt:** Writing – review and editing; supervision. **Dana Stearns:** Writing – review and editing; supervision. **Pilar Garcia Souto:** Software; formal analysis; methodology; supervision. **Anette Wu:** Writing – review and editing; conceptualization; resources; supervision; data curation; investigation; project administration.

## Supporting information


Data S1.

Figure S1.

Figure S2.

Data S2.
