# Supplementary figures and images for: Evaluating group dynamics through peer assessment during a global student collaboration of interprofessional healthcare education: A longitudinal study across 33 universities
Source: Anat Sci Educ. 2025 Mar 27;18(5):436–47. doi: 10.1002/ase.70026 (PMC12051087; doi:10.1002/ase.70026)

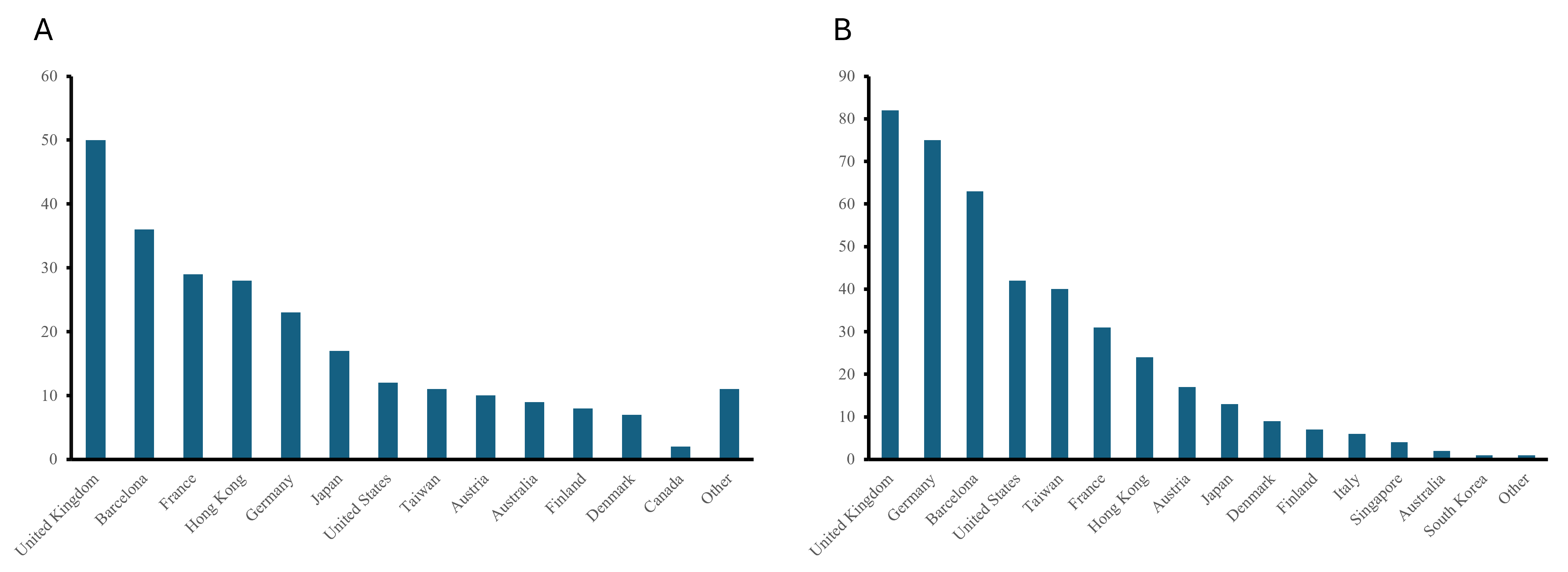

Supplement: Supplementary file 2 — Figure S1. [file ASE-18-436-s002.tif]

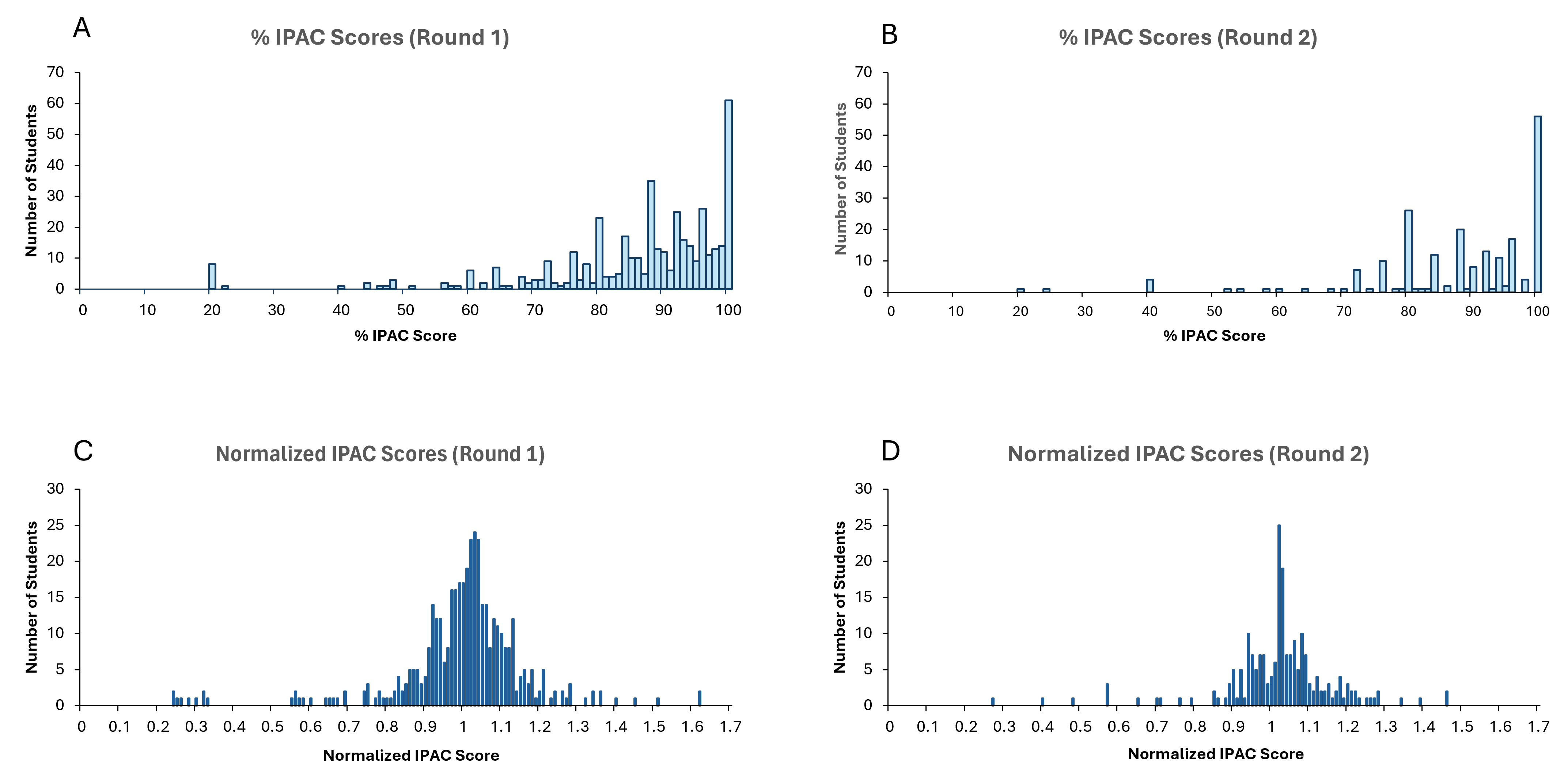

Supplement: Supplementary file 3 — Figure S2. [file ASE-18-436-s004.tif]
