# Supplementary material for: Evaluating group dynamics through peer assessment during a global student collaboration of interprofessional healthcare education: A longitudinal study across 33 universities
Source: Anat Sci Educ. 2025 Mar 27;18(5):436–47. doi: 10.1002/ase.70026 (PMC12051087; doi:10.1002/ase.70026)
Supplement: Supplementary file 4 — Data S2. [file ASE-18-436-s001.docx]

**Supplementary Table 1.** Survey and rating criteria used for peer feedback, adapted from the template provided within the IPAC system (UCL, 2015).

| 1 | What is your current degree of study?   - Medicine - MD/PhD - Dentistry - Nursing - Health Sciences - Others (please specify) |
| --- | --- |
| 2 | Which university are you currently studying in? |
| 3 | What is your age category?   - Under 20 - Between 20 and 25 - Over 25 |
| 4* | For each student in your group, provide a rating for the following in accordance to the rating criteria (1 lowest to 5 highest):   - Overall rating - Quality of work and insightful ideas - Punctuality and engagement in group meetings - Ability to work in a team and respecting others’ ideas - Leadership skills |
| 5* | For each student in your group, provide a comment about the student’s contribution to the development of ideas, executing group projects, engagement in group meetings, problem-solving skills and working as a team member. Answers are optional and kept anonymous. |

*The number of students in the group determines the number of times questions 4 and 5 are asked in the survey.

| **Rating** | **Criteria** |
| --- | --- |
| 5 | **Excellent** - The team member went above and beyond demonstrating high professional standards and commitment. |
| 4 | **Good** - The team member demonstrated a general good approach and performance in this category. There might be things that require improvement, but performance was above average and beneficial to the group. |
| 3 | **Satisfactory** - There is enough evidence of complying with this professional quality in general, though the team member does not excel at it. The student delivers what was expected of them but nothing more. |
| 2 | **Unsatisfactory** - There is some evidence of an attempt to comply with this professional quality, but the team member did no manage to achieve a satisfactory level. The student needed encouragement and was often unwilling to participate. The performance was not up to standard. |
| 1 | **Poor** - The performance of the student in this category is lacking. |

**Supplementary Table 2.** Comparison of percentage IPAC score distributions (%) from ICEP with previous studies (adapted from Garcia-Souto, July 2019).

|  | **Responses Received** | **Type** | **IPAC Distribution** | | | |
| --- | --- | --- | --- | --- | --- | --- |
|  |  |  | **Average** | **SD** | **Min** | **Max** |
| ICE Round 1 (2021-22) | 244 | % | 86.3 | 15.42 | 20 | 100 |
| ICE Round 2 (2021-22) | 154 | % | 86.9 | 14.5 | 20 | 100 |
| ICE Round 1 (2022-23) | 179 | % | 85 | 15.38 | 20 | 100 |
| ICE Round 2 (2022-23) | 55 | % | 91.4 | 11 | 40 | 100 |
| Garcia-Souto (2019) | 710 | % | 94.5 | 10.2 | 25 | 100 |
| Tucker (2014) | 603 F | % | 74.7 | 10.5 | - | - |
|  | 902 M |  | 73.4 | 12.6 | - | - |
| Northrup (2006) | 43 | % | 89.4 | 8.0 | 67.5 | 99.5 |
| Das (1998) | 64 | % | 79.7 | 17.3 | - | - |

**Supplementary Table 3.** Comparison of normalized IPAC score distributions (Norm) from ICEP with previous studies (adapted from Garcia-Souto, July 2019).

|  | **Responses Received** | **Type** | **IPAC Distribution** | | | |
| --- | --- | --- | --- | --- | --- | --- |
|  |  |  | **Average** | **SD** | **Min** | **Max** |
| ICE Round 1 (2021-22) | 244 | Norm | 1 | 0.173 | 0.25 | 1.51 |
| ICE Round 2 (2021-22) | 154 | Norm | 1 | 0.158 | 0.27 | 1.44 |
| ICE Round 1 (2022-23) | 179 | Norm | 1 | 0.166 | 0.24 | 1.62 |
| ICE Round 2 (2022-23) | 55 | Norm | 1 | 0.094 | 0.5581 | 1.24 |
| Garcia-Souto (2019) | 476 | Norm | 1 | 0.092 | 0.36 | 1.32 |
| Cheng (2000) | 53 | Norm | 1 | 0.044 | 0.86 | 1.1 |
| Conway (1993) | - | Norm | 1 | 0.094 | 0.76 | 1.19 |

**Supplementary Table 4.** Cronbach’s alpha values of peer assessment survey responses from each cohort.

| **Year** | **Evaluation Round** | **Cronbach’s alpha** |
| --- | --- | --- |
| 21−22 | Round 1 | 0.930 (Excellent) |
|  | Round 2 | 0.903 (Excellent) |
| 22−23 | Round 1 | 0.917 (Excellent) |
|  | Round 2 | 0.900 (Excellent) |
